# Association of adipokines, leptin/adiponectin ratio and C-reactive protein with obesity and type 2 diabetes mellitus

**DOI:** 10.1186/1758-5996-6-99

**Published:** 2014-09-16

**Authors:** Zaid Al-Hamodi, Molham AL-Habori, Ali Al-Meeri, Riyadh Saif-Ali

**Affiliations:** Department of Biochemistry and Molecular Biology, Faculty of Medicine and Health Sciences, University of Sana’a, PO Box 19065, Sana’a, Republic of Yemen

**Keywords:** Type 2 DM, Obesity, Adiponectin, Leptin, Leptin/Adiponectin ratio, hs-CRP

## Abstract

**Objective:**

Alterations in plasma adipokines and/or inflammatory parameters in Type 2 DM remain vague as to whether they are due to obesity and/or directly associated with the diabetic state. Our objective was to compare plasma adiponectin, leptin, leptin/adiponectin ratio (LAR) and hs-CRP in obese non-diabetic subjects and non-obese Type 2 DM patients, as well as determining the association of these adipokines with MetS and diabetes-related quantitative traits.

**Methods:**

In this study, 92 Yemeni male volunteers aged 25–60 years old were enrolled, 31 of whom were healthy subjects with BMI < 25 kg/m^2^ served as control; 30 non-diabetic obese subjects BMI ≥ 30 kg/m^2^ and FBG < 6.1 mmol/l; and 31 non-obese Type 2 DM with FBG > 7 mmol/l and BMI < 25 kg/m^2^.

**Results:**

Adiponectin was lower in obese subjects, with no differences between non-obese Type 2 DM patients and controls. In contrast, leptin, LAR and hs-CRP were higher in both obese subjects and non-obese Type 2 DM patients. Linear regression analysis showed adiponectin to be associated negatively with BMI, waist circumference, insulin, HOMA-β and HOMA-IR; whereas leptin, LAR and hs-CRP were associated positively with BMI, waist circumference, TG, FBG, insulin, HOMA-β and HOMA-IR. Moreover, adiponectin negatively correlated with leptin, LAR and hs-CRP; whereas leptin and LAR positively correlated with hs-CRP and with each other.

**Conclusion:**

Plasma adiponectin is not affected by diabetes *per se*, suggesting that its alterations in Type 2 DM may be due to obesity and may be an important link between adiposity, IR and Type 2 DM.

## Introduction

Type 2 DM is a heterogeneous disorder characterized by two interrelated metabolic defects: insulin resistance (IR) coupled with impaired β-cell function [1, 2]. In individuals with impaired glucose tolerance (IGT), numerous genetic, host-related, and environmental factors contribute to the progression of IR to Type 2 DM. Obesity, and especially visceral adipose tissue accumulation, increases the risk of developing Type 2 DM [3]. The greater risk of Type 2 DM in the obese can partly be explained by changes in adipose tissue function [4, 5]. In the last decade, a hypothesis was proposed to associate the pathogenesis of Type 2 DM with a state of subclinical chronic inflammation [6, 7]. Epidemiological studies have demonstrated an increase in plasma levels of inflammatory markers such as C-reactive protein (CRP), interleukin-6 (IL-6) and tumour necrosis factor-α (TNF-α) in patients with metabolic syndrome (MetS) and also in those with clinically overt Type 2 DM [8]. Chronically elevated levels of these biomarkers promote IR in skeletal muscle and endothelial dysfunction [9] as well as in adipose and other tissues, thereby increasing the risk for Type 2 DM [10].

Adiponectin is almost exclusively secreted by adipocytes and appears to acts as a hormone with anti-inflammatory and insulin-sensitizing properties *in vitro* and in animal models and may also promote *β*-cell function and survival [11, 12]. Adiponectin is almost unique among adipokines because its systemic levels decrease in obesity, whereas most other adipokines (in particular the pro-inflammatory adipokines) are released in larger amounts with cell size [12, 13]. Adiponectin levels are decreased in cardiovascular disease and several metabolic disorders including obesity, inflammatory states, IR, and Type 2 DM [14, 15]. Several clinical studies have demonstrated hypoadiponectinemia correlates with the development of IR and Type 2 DM [16–19]. The majority of these studies show the adiponectin level in Type 2 DM to be often reduced, even in the relative early stage of this condition, such as at time of diagnosis [20, 21]. A range of prospective studies demonstrated that high levels of circulating adiponectin are associated with low risk of Type 2 DM even after adjustment for multiple factors that could confound this relationship [22–25].

Leptin is a peptide hormone mainly secreted by white adipose tissue [26]. Its expression and release are increased in large adipocytes even after normalization for fat cell volume so that its regulation shows similarities to the regulation of pro-inflammatory immune-mediators in adipocytes [13]. It acts on the hypothalamus, leading to decreased appetite and increased energy expenditure, thereby regulating body weight [27]. Furthermore, leptin has a number of other activities, including regulation of endocrine function, reproduction and immunity [26, 28]. In humans, obesity is associated with high circulating leptin levels probably reflecting a state of leptin resistance, i.e. impaired leptin signalling and action. This state could interfere with the physiological relationship between leptin and β-cell function and promote the development of IR and Type 2 DM [29]. Previous epidemiological studies investigating the association between circulating leptin levels and incident of Type 2 DM yielded discrepant results [16, 30].

It remains unclear whether the observed alterations in plasma adipokines and/or inflammatory parameters in Type 2 DM are due to excess adipose tissue mass and/or directly associated with the diabetic state [31, 32]. We hypothesize that altered plasma adipokine and/or inflammatory factor levels are related to the obese state and are not prevalent in non-obese Type 2 DM. The present study compares plasma adipokines and inflammatory markers between obese non-diabetic individuals and non-obese Type 2 DM patients versus non-obese, normoglycemic controls; as well as evaluating the association of adipokines and inflammatory markers with metabolic syndrome and diabetes-related quantitative traits. We also examined the Leptin/Adiponectin ratio (LAR) in view of previous reports suggesting that LAR being a better indicator of IR than the single adipokines [33].

## Research design and methods

### Study subjects

For this study, a total of 92 Yemeni men subjects aged 25–60 years were recruited from Al-Thwara Hospital, Sana’a. Thirty one were healthy non-obese, normoglycaemic subjects with a BMI < 25 kg/m^2^ served as controls; 30 obese non-diabetic subjects with BMI ≥ 30 kg/m^2^ and FBG *<* 6.1 mmol/L; and 31 non-obese Type 2 DM patients with blood glucose > 7 mmol/l and BMI < 25 kg/m^2^. Obese Type 2 DM patients were excluded in order to examine the role of diabetes per se. In general, subjects with acute or chronic infections, severe medical conditions (malignancy, renal failure, liver cirrhosis, connective tissue disease, and chronic congestive heart failure) were excluded from the study. The study protocol was approved by the institutional review board (IRB) of the Faculty of Medicine and Health Sciences, Sana’a University. Informed consent was obtained from all individuals after explaining the purpose and nature of the study.

Blood pressure (BP) measurements were taken from each patient’s right arm in the seated position by using an Omron IntelliSense Automatic Blood Pressure Monitor after 10 min of rest in a quiet room. Two to three successive BP readings were obtained at 5 minutes intervals and averaged. Body weight and height were measured without shoes in the morning, and BMI was computed as weight in kilograms (kg) divided by height in meters squared (m^2^). Waist circumference was measured midway between the lower rib margin and the superior iliac spine at the end of gentle expiration in a standing position. Fasting venous blood (6 ml) was collected from each subject after 12 hours fast and immediately taken into 2 labelled test tubes, sodium fluoride (for glucose measurement) and plain tubes for other biochemical investigations. The plain tubes were centrifuged for 15 minutes at 2500-3000 × g within 30 minutes of blood collection and the serum from each sample was separated into four micro tubes and immediately kept at -20°C until analysis. Plasma glucose was determined immediately after centrifugation.

### Biochemical analyses

Serum triglyceride (TG), HDL-cholesterol (HDL-c), and plasma glucose (FPG) were manually measured by their respective kits (Human Company, Germany). Insulin was measured by Electrochemiluminescence immunoassay (ECL) on Elecsys autoanalyzer (Roche Diagnostics, Germany). Insulin resistance and Insulin sensitivity (IS) were calculated using the Homeostasis Model Assessment (HOMA 2) Calculator v2.2 which is available from Oxford Centre for Diabetes, Endocrinology and Metabolism. ELISA kits were used to measure serum adiponectin (R&D Systems, USA), serum leptin and hs-CRP (DRG Diagnostics, Germany).

### Statistical analyses

The results were analyzed by the Social Package of Statistical Science (SPSS) software version 11.5 (SPSS Inc., Chicago, IL, USA). Age, BMI, waist circumference, systolic blood pressure (SBP), diastolic blood pressure (DBP), TG, HDL-c, FBG, insulin, IS, HOMA-IR, adiponectin, leptin, LAR and hs-CRP were log transformed because they were not normally distributed. These parameters means and 95% confidence intervals were transformed back and reported as geometric means. ANOVA test was used to describe the mean differences among groups of study. General linear model, univariate analyses (ANCOVA) adjusted for age was used to assess the differences of adiponectin, leptin, LAR and hs-CRP between obese non-diabetic, non-obese Type 2 DM and normal subjects. The association of adiponectin, leptin, LAR and hs-CRP with metabolic syndrome and diabetes-related qualitative traits: BMI, waist circumference, SBP, DBP, TG, HDL-c, FBG, insulin, IS, HOMA-β and HOMA-IR (dependent variables) were analyzed by hierarchical linear regression adjusted for age in a combined group: normal and obese non-diabetic subjects. The interrelationship between adiponectin, leptin, LAR and hs-CRP in non-diabetic subjects were evaluated by linear regression controlled for age.

## Results

The demographic and biochemical parameters of the subjects are shown in Table 1. The adiponectin, leptin, LAR and hs-CRP assessed by general linear model (univariate) are shown in Table 2. Serum levels of adiponectin were significantly lower in obese non-diabetic subjects compared to control and non-obese Type 2 DM subjects (p = 0.002, 0.006 respectively), with no differences in adiponectin levels between non-obese Type 2 DM subjects and controls. In contrast, serum leptin levels and LAR were higher in both obese non-diabeic subjects (p = 3.6 × 10^-30^, 1.1 × 10^-26^) and non-obese Type 2 DM (p = 2.8 × 10^-19^, 2.9 × 10^-12^, respectively) as compared to control subjects. In addition, serum leptin levels and LAR in obese subjects were higher with respect to non-obese Type 2 DM subjects (p = 2.7 × 10^-4^, 1.4 × 10^-7^, respectively). Similarly, serum hs-CRP levels were higher in both obese subjects and non-obese Type 2 DM patients as compared to control subjects (p = 4.3 × 10^-6^, 8.2 × 10^-10^, respectively). Unlike that of leptin and LAR, serum hs-CRP levels were higher in non-obese Type 2 DM than those in obese subjects (p = 0.019).

**Table 1:** **Demographic and biochemical parameters among normal, non-diabetic obese and non-obese type 2 diabetes mellitus groups**

Parameters	Normal (n = 31)	Non-diabetic obese (n = 30)	Non-obese T2DM (n = 31)
**Age (years)**	31.3 (27.8-35.2)	28.4 (26.6-32.4)	45.2 (39.8-51.3)
**p-value**		^a^0.44	^a^ ***4.5 × 10*** ^***-5***^, ^b^ ***4.0 × 10*** ^***-5***^
**BMI (kg/m** ^**2**^ **)**	20.6 (19.7-21.5)	34.0 (32.5-35.5)	22.3 (21.1-23.7)
**p-value**		^a^ ***5.1 × 10*** ^***-9***^	^a^0.53, ^b^ ***5.1 × 10*** ^***-9***^
**Waist circumference (cm)**	75.1 (72.7-77.6)	90.2 (88.0-92.5)	71.3 (69.2-73.5)
**p-value**		^a^ ***5.1 × 10*** ^***-9***^	^***a***^ ***0.037***, ^b^ ***5.1 × 10*** ^***-9***^
**DBP (mmHg)**	75.5 (72.5-78.7)	85.2 (82.8-87.6)	83.5 (79.8-87.3)
**p-value**		^a^ ***4.9 × 10*** ^***-5***^	^a^ ***0.00096,*** ^b^0.74
**SBP (mmHg)**	113 (109–117)	126 (123–130)	123 (116–130)
**p-value**		^a^ ***2.3 × 10*** ^***-4***^	^a^ ***0.009*** *,* ^b^0.56
**Triglyceride (mmol/l)**	1.5 (1.2-1.8)	2.1 (2.0-2.2)	2.0 (1.8-2.2)
**p-value**		^a^ ***0.0009***	^a^ ***0.005***, ^b^0.88
**HDL-c (mmol/l)**	0.9 (0.8-1.0)	0.8 (0.7-0.9)	0.7 (0.6- 0.8)
**p-value**		^a^0.23	^a^ ***0.01***, ^b^0.34
**FBG (mmol/l)**	5.4 (5.1-5.8)	5.7 (5.5-6.0)	9.7 (8.3-11.4)
**p-value**		^a^0.66	^a^ ***5.1 × 10*** ^***-9***^ ***,*** ^b^ ***5.2 × 10*** ^***-9***^
**Insulin (pmol/l)**	44.5 (37.2-53.2)	130 (112–151)	70.1 (54.3-90.5)
**p-value**		^a^ ***5.1 × 10*** ^***-9***^	^a^ ***0.004, 5.9 × 10*** ^***-5***^
**HOMA-β**	73.2 (62.3-85.9)	135 (119–154)	33.7 (23.0-49.2)
**p-value**		^a^ ***0.0007,***	^a^ ***3.2 × 10*** ^***-*5**^ ***,*** ^b^ ***5.1 × 10*** ^***-9***^
**Insulin Sensitivity (%)**	118 (99–142)	40.8 (35.2- 47.3)	62.4 (49.0-79.3)
**p-value**		^a^ ***5.1 × 10*** ^***-9***^	^a^ ***1.6 × 10*** ^***-5***^ ***, 0.006***
**HOMA-IR**	0.9 (0.7-1.1)	2.5 (2.2-2.9)	1.7 (1.3-2.2)
**p-value**		^a^ ***5.1 × 10*** ^***-9***^	^a^ ***4.9 × 10*** ^***-5***^ ***, 0.005***

Result presented as geometric mean and 95% confidence interval of mean; ^a^vs. normal group; ^b^vs. non-diabetic obese group evaluated by ANOVA.

**Table 2:** **Comparison of adiponectin, leptin, leptin/adiponectin ratio (LAR) and hs-CRP between normal, non-diabetic obese and non-obese type 2 DM groups**

Parameters	Normal (n = 31)	Non-diabetic obese (n = 30)	Non-obese T2DM (n = 31)
**Adiponectin (IU/ml)**	25 (20.6-30.2)	16.3 (13.3-19.9)	25.8 (20.6-32.2)
**p-value**		^a^ ***0.002***	^a^0.83, ^b^ ***0.006***
**Leptin (ng/ml)**	14 (12–17)	141 (116–172)	78 (63–96)
**p-value**		^a^ ***3.6 × 10*** ^***-30***^	^*a*^ ***2.8 × 10*** ^***-19***^ ***,*** ^*b*^ ***2.7 × 10*** ^***-4***^
**Leptin/Adiponectin Ratio**	0.59 (0.36-0.85)	8.21 (6.78-9.90)	3.31 (2.61-4.15)
**p-value**		^a^ ***1.1 × 10*** ^***-26***^	^***a***^ ***2.9 × 10*** ^***-12***^ ***,*** ^***b***^ ***1.4 × 10*** ^***-7***^
**hs-CRP (U/ml)**	15.2 (14.7-15.7)	17.0 (16.5-17.7)	18.2 (17.6-19.0)
**p-value**		^a^ ***4.3 × 10*** ^***-6***^	^a^ ***8.2 × 10*** ^***-10***^, ^b^ ***0.019***

Results presented as geometric mean and 95% confidence interval of mean adjusted for age; ^a^vs. normal group, ^b^vs. non-diabetic obese group evaluated by univariate (General Linear Model).

The association of the inflammatory related variables (adiponectin, leptin, LAR and hs-CRP) with metabolic syndrome and diabetes-related qualitative traits was evaluated in the combined group; normal and obese non-diabetic subjects (Table 3). Serum levels of adiponectin associated with increased HDL-c and IS, and decreased BMI, waist circumference, insulin, HOMA-β and HOMA-IR. In contrast, serum leptin levels and LAR associated with decreased IS, and increased BMI, waist circumference, DBP, SBP, TG, FBG, insulin, HOMA-β and HOMA-IR. On the other hand, hs-CRP associated with decreased HDL-c and IS, and increased BMI, waist circumference, DBP, SBP, TG, FBG, insulin, HOMA-β, and HOMA-IR.

**Table 3:** **Association of adiponectin, leptin, leptin/adiponectin ratio (LAR) and hs-CRP with metabolic syndrome and diabetic parameters among non-diabetic subjects (n = 61)**

Metabolic syndrome parameters	Adiponectin (IU/ml)	Leptin (ng/ml)	Leptin/Adiponectin Ratio	hs-CRP (U/ml)
**BMI (kg/m2)**	-0.213 ***(0.0007)***	0.049 ***(1.1 × 10*** ^***-11***^ ***)***	0.208 ***(3.2 × 10*** ^***-9***^ ***)***	3.256 ***(2.2 × 10*** ^***-10***^ ***)***
**Waist circumference (cm)**	-0.368 ***(9.5 × 10*** ^***-5***^ ***)***	0.070 ***(1.6 × 10*** ^***-9***^ ***)***	0.706 ***(8.2 × 10*** ^***-7***^ ***)***	5.302 ***(2.1 × 10*** ^***-10***^ ***)***
**DBP (mmHg)**	-0.032 (0.789)	0.044 ***(0.001)***	0.481 ***(0.003)***	2.955 ***(0.002)***
**SBP (mmHg)**	0.009 (0.916)	0.032 ***(0.001)***	0.309 ***(0.01)***	1.685 ***(0.015)***
**Triglyceride (mmol/l)**	-0.004 (0.208)	0.002 ***(0.0001)***	0.014 ***(0.003)***	0.127 ***(2.8 × 10*** ^***-6***^ ***)***
**HDL-c (mmol/l)**	0.005 ***(0.034)***	-0.0003(0.203)	-0.006 (0.061)	-0.035 ***(0.050)***
**LDL-c (mmol/l)**	- 0.011 (0.157)	0.003 ***(0.0016)***	0.024 ***(0.032)***	0.117 (0.074)
**FBG (mmol/l)**	- 0.005 (0.468)	0.003 ***(0.003)***	0.023 ***(0.02)***	0.143 ***(0.016)***
**Insulin (pmol/l)**	-1.163 ***(0.018)***	0.285 ***(1.1 × 10*** ^***-6***^ ***)***	0.342 ***(5.3 × 10*** ^***-6***^ ***)***	22.91 ***(1.6 × 10*** ^***-8***^ ***)***
**HOMA-β**	-1.152 ***(0.006)***	0.132 ***(0.01)***	0.143 ***(0.002)***	14.44 ***(6.3 × 10*** ^***-5***^ ***)***
**Insulin Sensitivity (%)**	1.583 ***(0.001)***	-0.279 ***(2.7 × 10*** ^***-5***^ ***)***	-0.248 ***(0.0001)***	-20.3 ((***4.3 × 10*** ^***-6***^)
**HOMA-IR**	-0.021 ***(0.021)***	0.006***(6.3 × 10*** ^***-7***^ ***)***	0.344 (***4.6 × 10*** ^***-6***^)	0.436 (***1.3 × 10*** ^***-8***^)

Results presented represent b values (p values) assessed by linear regression adjusted for age; (b) coefficient for the relationship between the dependent variables (adiponectin, leptin, LAR and hs-CRP) and the independent variables (metabolic syndrome and diabetic parameters).

The interrelationship between adiponectin, leptin, LAR and hs-CRP parameters was further assessed in the combined group; normal and obese non-diabetic subjects as shown in Table 4. Serum adiponectin levels negatively correlated with leptin, LAR and hs-CRP (r^2^ = -0.216, p = 0.002; r^2^ = -0.268, p = 0.0001; r^2^ = -0.098, p = 0.006). In contrast, serum leptin levels and LAR positively correlated with hs-CRP (r^2^ = -0.528, p = 5.8 × 10^-11^; r^2^ = -0.527, p = 6.7 × 10^-11^ respectively), and with each other (r^2^ = -0.668, p = 1.4 × 10^-14^).

**Table 4:** **Correlation between adiponectin, leptin, leptin/Adiponectin ratio and hs-CRP parameters among non-diabetic subjects (n = 61)**

Parameters	Adiponectin (IU/ml)	Leptin (ng/ml)	Leptin/Adiponectin ratio	hs-CRP (U/ml)
**Adiponectin (IU/ml)**	**1**	-0.216 ***(0.002)***	-0.268 ***(0.0001)***	-0.098 ***(0.006)***
**Leptin**		1	0.668 ***(1.4 × 10*** ^***-14***^ ***)***	0.528 ***(5.8 × 10*** ^***-11***^ ***)***
**Leptin/Adiponectin**			1	0.527 ***(6.7 × 10*** ^***-11***^ ***)***

Result presented as adjusted r^2^ (P-value) assessed by Linear regression, adjusted for age.

## Discussion

The results presented in our study shows plasma adiponectin not to be affected by diabetes *per se* whereby adiponectin was significantly lower in obese individuals as compared to both control and non-obese Type 2 DM, with no difference in adiponectin between non-obese Type 2 DM and control group; thus suggesting that the often reported alterations in plasma adiponectins in Type 2 DM [32, 34, 35] may be due to excess adipose tissue mass/ obesity. In accordance with our results, a recent finding suggested that adiponectin levels in patients with Type 2 DM seem to be more associated with obesity and less with diabetes [36]. In general most of the Type 2 DM patients included in those studies were obese. To differentiate between the impact of obesity and type 2 diabetes on the altered plasma adipokine and/or inflammatory profiles, a recent study compares basal plasma adipokines and inflammatory markers between obese and non-obese type 2 diabetes patients versus non-obese, normoglycemic controls and conclude that the observed alterations in these parameters in obese type 2 diabetes patients as opposed to non-obese Type 2 DM are attributed to the greater adipose tissue mass, and not necessarily to the presence of the type 2 diabetic state [37]. Taking into account the ethnical variation our study was designed to assess the association of these adipokines in Yemeni Type 2 DM patients as well as highlighting the impact of diabetes per se.

Our results also show adiponectin to be associated with metabolic syndrome and diabetes-related qualitative traits, whereby adiponectin was associated with increased HDL-c and IS; and decreased BMI, waist circumference, insulin, HOMA-IR and HOMA-β; which is in line with previous studies [38–40]. Many studies suggest that adiponectin is an important regulator of insulin sensitivity and glucose homeostasis, with several reports confirming an inverse relationship between insulin resistance and plasma adiponectin levels [19, 23, 41]. The evidence that insulin may have direct effect on adiponectin gene expression and adiponectin concentrations *in vitro* [42] may infer that the higher levels of insulin in insulin-resistant subjects may down-regulate levels of adiponectin [43]. However, the inverse association between adiponectin and IR in our results and others [39, 40] may possibly be mediated not only by insulin but also by inflammatory cytokines [44]. Moreover, the positive association between adiponectin and HDL-c is in support of earlier studies in the general population [38] and obese subjects [45], suggesting that adiponectin plays a role in HDL metabolism.

Unlike adiponectin, serum leptin levels and LAR were higher in both obese individuals and non-obese Type 2 DM patients with respect to the control group. In addition, serum leptin levels and LAR in obese subjects were higher compared to non-obese Type 2 DM. Our results are consistent with previous studies showing circulating leptin levels to be higher in obese individuals and patients with MetS [40, 46]. Earlier reports showed circulating leptin concentrations to be elevated in obese subjects in proportion to the degree of adiposity [46] and positively correlated with body fat mass, despite anti-obesity actions of leptin [47]. *In vitro* studies also indicated that leptin secretion was significantly greater in enlarged adipocytes [13].

Our results show that adiponectin levels and that of leptin and LAR exhibit opposing and variable correlations with obesity, IR and the components of the metabolic syndrome; whereby leptin levels and LAR were associated with decreased IS and increased BMI, waist circumference, SBP, DBP, TG, FBG, insulin, HOMA-IR and HOMA-β. These findings are in agreement with previous studies [39, 40, 48]. High leptin levels generally associated with high insulin levels could be partially explained by a state of leptin resistance such that chronically elevated leptin levels in obesity may result in decreased responsiveness of the receptor system in pancreatic β cells, leading to increased insulin secretion. The resulting hyperinsulinaemia in turn could exacerbate obesity and further increase leptin levels, resulting in a positive feedback loop that could promote the development of diabetes [24, 29]. Accordingly, our results show a close relationship between insulin and leptin levels in non-diabetic obese subjects.

Our results also show an inverse association between leptin and adiponectin which highlights the recent findings that indicated that the two adipokines interact with each other in the modulation of Type 2 DM risk [24]. Perhaps this association could be secondary to the inverse relation of these adipokines with fat mass; direct relation of leptin; and inverse relation of adiponectin. In obesity where plasma adipokine levels are reduced, this can be used as a marker of insulin resistance and predict the development of Type 2 DM and atherosclerosis [41]. The observed increase in LAR in both obese individuals and non-obese Type 2 DM as compared to the increase in HOMA-IR may suggest that LAR could be a useful index for insulin resistance. This is in line with recent suggestion that LAR could serve as a useful index for insulin resistance in clinical practice and a good indicator for assessing the effectiveness of anti-diabetic therapy [33] as well as a potential atherogenic index in obese Type 2 DM patients [49].

Since chronic inflammation is likely to play a role in the pathogenesis of Type 2 DM, we also examined the association between adipocytokines and hs-CRP, an inflammatory marker that is related to both MetS and cardiovascular events; and found that adiponectin was inversely correlated with hs-CRP levels, which is in agreement with earlier studies [50] inferring that adiponectin may be an important link between adiposity, inflammation and Type 2 DM [41]. Therefore, adiponectin may directly or indirectly affect CRP levels through modulating inflammatory cascades [35]. Moreover, we found a positive association between serum leptin levels and hs-CRP, which can be explained in part by the fact that adipocytes by serving as a common source for both leptin and inflammatory cytokines contribute to CRP synthesis. Leptin may directly induce production of IL-6, resulting in up-regulation of hepatic CRP production [26].

Serum hs-CRP levels were higher in both obese individuals and non-obese Type 2 DM compared to control group. Unlike that of leptin and LAR, serum hs-CRP levels were higher in non-obese Type 2 DM than that in obese subject, which is concordant with several cross-sectional studies showing an increase of CRP levels in patients with diabetes [32, 35, 51, 52]. Epidemiological studies have demonstrated an increase in plasma levels of inflammatory markers such as CRP, IL-6 and TNF-α in patients with MetS and also in those with clinically overt Type 2 DM [8]. C-reactive protein also appears to progressively increase when glucose metabolism deteriorates, as evident in subjects with IFG and Type 2 DM [34, 53]. The significant increment of serum hs-CRP levels in our non-obese Type 2 DM, and the positive correlation between hs-CRP and HOMA-IR suggest that chronic inflammation may play a role in the development of insulin resistance and Type 2DM. The pathophysiological mechanisms linking obesity to elevated levels of CRP are well recognized [4]. In a recent systematic review and meta-analysis, obesity was strongly associated with elevated levels of CRP in all populations observed [54]. The ongoing debate regarding the association among chronic inflammatory states; insulin resistance and obesity provide a conflicting hypothesis that chronic inflammatory state originates from obesity and drives the insulin resistant condition. Moreover, hs-CRP in non-diabetic subjects was associated with decreased HDL-c and IS and increased BMI, waist circumference, SBP, DBP, TG, FBG, insulin, HOMA-IR and HOMA-β, which is in line with previous studies demonstrating a significant correlation between CRP levels and features of the MetS; including adiposity, hyperinsulinemia and IR [55, 56]. The observed strong association of fasting insulin with CRP concentration has previously been demonstrated [57].

There were some limitations in this study; first, the study is hospital based and all subjects were male therefore the findings may not generally be applicable to the overall population. Second, the sample size was small, limiting its statistical power for detecting associations. Third, the β–cell function was evaluated using the homeostasis model (HOMA- β and HOMA-IR) as there is no “gold standard” method to evaluate β-cell function/mass *in vivo*. Nevertheless, this study represents a simultaneous investigation of relationships between serum adipokine profile and inflammatory markers in Yemeni non-obese Type 2 DM patients.

In conclusion, plasma adiponectin was not affected by diabetes *per se*, suggesting that the often reported alterations in plasma adiponectins in Type 2 DM may be due to excess adipose tissue mass/ obesity. Adiponectin and leptin exhibited opposing and variable correlations with obesity, IR and the components of the metabolic syndrome. In addition, LAR was strongly correlated with the HOMA-IR supporting findings that LAR could be a potential marker of IR. The inverse association between adiponectin and hs-CRP infers that adiponectin may be an important link between adiposity, inflammation and Type 2 DM. Moreover, the significant increment of hs-CRP in non-obese Type 2 DM, and its positive correlation with HOMA-IR suggest that chronic inflammation may play a role in the development of insulin resistance and Type 2DM.
